# Silver Carboxylate as an Antibiotic-Independent Antimicrobial: A Review of Current Formulations, *in vitro* Efficacy, and Clinical Relevance

**DOI:** 10.18103/mra.v10i12.3388

**Published:** 2022-12-21

**Authors:** Makena Mette, William Connolly, Neel Vishwanath, Sai Allu, Colin Whitaker, Benjamin K. Stone, Valentin Antoci, Christopher T. Born, Dioscaris R. Garcia

**Affiliations:** 1 Warren Alpert Medical School of Brown University, Providence, RI; 2 Weiss Center for Orthopaedic Trauma Research, Rhode Island Hospital, Providence, RI; 3 Department of Orthopaedic Surgery, Warren Alpert Medical School of Brown University, Providence, RI

## Abstract

The increasing prevalence of multi-drug resistant pathogens has led to a renewed focus on the use of silver as an antibiotic-independent antimicrobial. Unfortunately, the use of many silver formulations may be limited by an uncontrolled release of silver with the potential for significant cytotoxic effects. Silver carboxylate (AgCar) has emerged as an alternative formulation of silver with the potential to mitigate these concerns while still displaying significant bactericidal activity. This article reviews the efficacy of silver carboxylate formulations as a promising novel antibiotic-independent antimicrobial.

This study was conducted through a search of five electronic databases (PubMed, Embase, MEDLINE, Cochrane Library, and Web of Science) for relevant studies up to September 2022. Searches were conducted for types of “silver carboxylate” formulations. Sources were compiled based on title and abstract and screened for inclusion based on relevance and study design. A review of the antimicrobial activity and cytotoxicity of silver carboxylate was compiled based on this search.

Current body of data suggests that silver carboxylate shows promise as an emerging antibiotic-independent antimicrobial, with significant bactericidal effects while minimizing cytotoxicity. Silver carboxylate addresses several of the limitations of more primitive formulations, including controlled dosing and fewer negative effects on eukaryotic cell lines. These factors are concentration-dependent and largely rely on the vehicle system used to deliver it. Although several silver carboxylate-based formulations like titanium dioxide/polydimethylsiloxane (TiO_2_/PDMS) matrix-eluting AgCar have shown promising results *in vitro*, and could potentially be utilized independently or in conjunction with current and future antimicrobial therapies, there is a need for further *in vivo* studies to validate their overall safety and efficacy profile.

## Introduction

Post-operational surgical site infections (SSIs) account for 20–30% of hospital acquired infections (HAIs).^1,2^ These infections have a high morbidity rate and often require revision surgeries which burden the healthcare system with increased costs.^1,3,4^ With the increased prevalence of multidrug-resistant pathogens,^5–7^ common antibiotics are becoming less effective at inhibiting pathogen growth, necessitating the development of alternative antimicrobial treatments.^6,8^ This need for alternative antimicrobial therapies is additionally heightened by the challenges of biofilms and persister cells. Rather *et al.* (2021) discuss the threat of biofilms and the “multifold microbial resistance exhibited by microorganisms dwelling within” them.^9^ Both Fisher *et al.* (2017) and Roy *et al*. (2018) also discuss the threat of persister cells, which also enhance the rising threat of antimicrobial resistance (AMR).^10,11^

While antimicrobial resistance, along with biofilms, and persister cells, constitute an enormous threat to modern medicine, several avenues of combatting this resistance are currently being investigated. Pang *et al.* (2019) discussed the development of new antibiotics for treating *Pseudomonas aeruginosa*, including doripenem, plazomicin, and protein epitope mimetic (PEM) POL7001; however, they acknowledged the limitations and time-consuming aspects of such an approach.^12^ Coates *et al.* (2020) reviewed combination antibiotic therapies, concluding that “antibiotic combination therapy, exploiting synergies, old-drug rejuvenation and resistance reduction could provide the solution to AMR”.^13^ However, it is unlikely that these strategies would be able to entirely eliminate the threat of AMR.^13^ In respect to biofilms, several advances in combatting their formation and resistance are currently being explored. Rather *et al.* (2021) elucidated several of these potential therapies, including different physical, chemical, and biological control strategies.^9^ Cascioferro *et al.* (2021) also performed a review of small molecule inhibitors of biofilm formation specifically for methicillin-resistant *Staphylococcus aureus* (MRSA) and concluded that while significant progress has been made, further research is required to address MRSA biofilm formation.^14^ Potentially more promising, more recent micro- and nanotechnology developments have been explored by Rao *et al*. (2021).^15^ These technologies have the advantage of having both their own antimicrobial traits and also the ability to potentially act as antimicrobial delivery systems, acting synergistically to combat biofilms and persister cells.^15^ In respect to persister cells, Song and Wood (2020) studied the use of indoles and substituted indoles, which had originally been thought to increase persistence, as potential effective inhibitors.^16^ Kim *et al.* (2018) also reviewed potential persister cell treatments, specifically for MRSA, including targeting of growth-independent targets and augmentation of uptake and accessibility of current antibiotics.^17^ Other antimicrobial resistance treatment agents are also in development, including antimicrobial peptides (AMPs).^18^ Peng *et al.* (2021) have explored the use of AMPs as an alternative to antibiotics, showing that a modified form of cecropin, called C18, has a multimodal antimicrobial effect on MRSA disrupting bacterial membranes as well as down-regulating virulence factor genes.^18^ However, this study did not study the cytotoxicity profile of C18 as it relates to human cell lines.

As alternative next-generation antimicrobials are being developed, certain traits are important to consider for their viability: By potentially employing synergistic capabilities for augmenting antibiotic efficacy (as discussed in Kim *et al.* (2018)) or increasing antibiotic shelf-life, new antimicrobials could be even more effective. Preventing microbial adherence through compatibility with commonly used coatings and materials, such as implant materials, sutures, and surgical dressings, could also allow for a wider applicability of new antimicrobials.^19–23^ Augmentation of penetration into areas where persister cells and biofilms thrive due to inaccessibility, such as pilosebaceous glands where pathogens like *Cutibacterium acnes* reside, would also enhance efficacy.^20,24,25^ Lastly, as previously discussed, the ability of the antimicrobial agent to be effective against persister cells and biofilm formation, in addition to general antibiotic resistance, is key to addressing the growing problem.

While several of the previously discussed methods of addressing antimicrobial resistance are nascent in their discovery and development, silver has historically been used as an antimicrobial agent with potential to overcome bacterial resistance profiles due to its multimodal method of antisepsis.^26,27^ However, concerns exist regarding its cytotoxicity against human cells.^26,28^ To address these concerns, silver carboxylate, a more novel formulation, has emerged as a likely safer and more efficacious alternative, especially as it potentially possesses several of the key novel antimicrobial traits mentioned previously.^20,22,23,29–32^ Several studies on the formulation of silver matrices, antimicrobial activity, and cytotoxicity are currently being conducted to evaluate silver carboxylate as an effective clinical tool.^22,23^

Historically, silver has been utilized as an antimicrobial in varying formulations, each with its own strengths and weaknesses.^26^ Ionic silver, silver nanoparticles (AgNPs), colloidal silver, and silver nitrate are all potent antimicrobial agents; however, these formulations are limited by an uncontrolled release of silver ions, that can lead to cytotoxicity.^26^ Newer formulations of silver, such as silver sulfadiazine, silver oxide, and AQUACEL ^®^ Ag SURGICAL Cover Dressing (ConvaTec, Berkshire, UK) improve upon the cytotoxic properties of the earlier silver formulations, but still have limitations, such as a high cost of treatment, and comparable alternatives for treatment with smaller side effect profiles.^26^

Silver also has significant potential for the treatment of multidrug-resistant (MDR) pathogens. A proprietary formulation of silver carboxylate (AgCar) that has undergone extensive validation^20,22,23,30^ has emerged as a promising antimicrobial silver formulation due to a matrix chemistry that allows for a controlled and predictable loading and release of silver carboxylate.^29–31^ This modification has been reported to result in an increase in duration of action, a decrease in toxicity and is a potential solution to maintaining longer bactericidal activity with an improved safety profile.^29–31^ To date, there is no literature that reviews the current uses, efficacy, and limitations of silver carboxylate. Given the growing body of literature supporting AgCar as a novel antimicrobial, we aim to provide a comprehensive analysis of current research on silver carboxylate, its applications as a clinical antimicrobial, and its potential for an improved cytotoxicity profile.

### Literature Search

A systematic search of PubMed, MEDLINE, Embase, Cochrane Library, and Web of Science was performed from the earliest available article to September 2022. Searches were conducted using the search term “silver carboxylate.” This resulted in a total of 148 articles, which were then screened for inclusion by authors based on title and abstract.

### Study Selection

**Figure 1:**
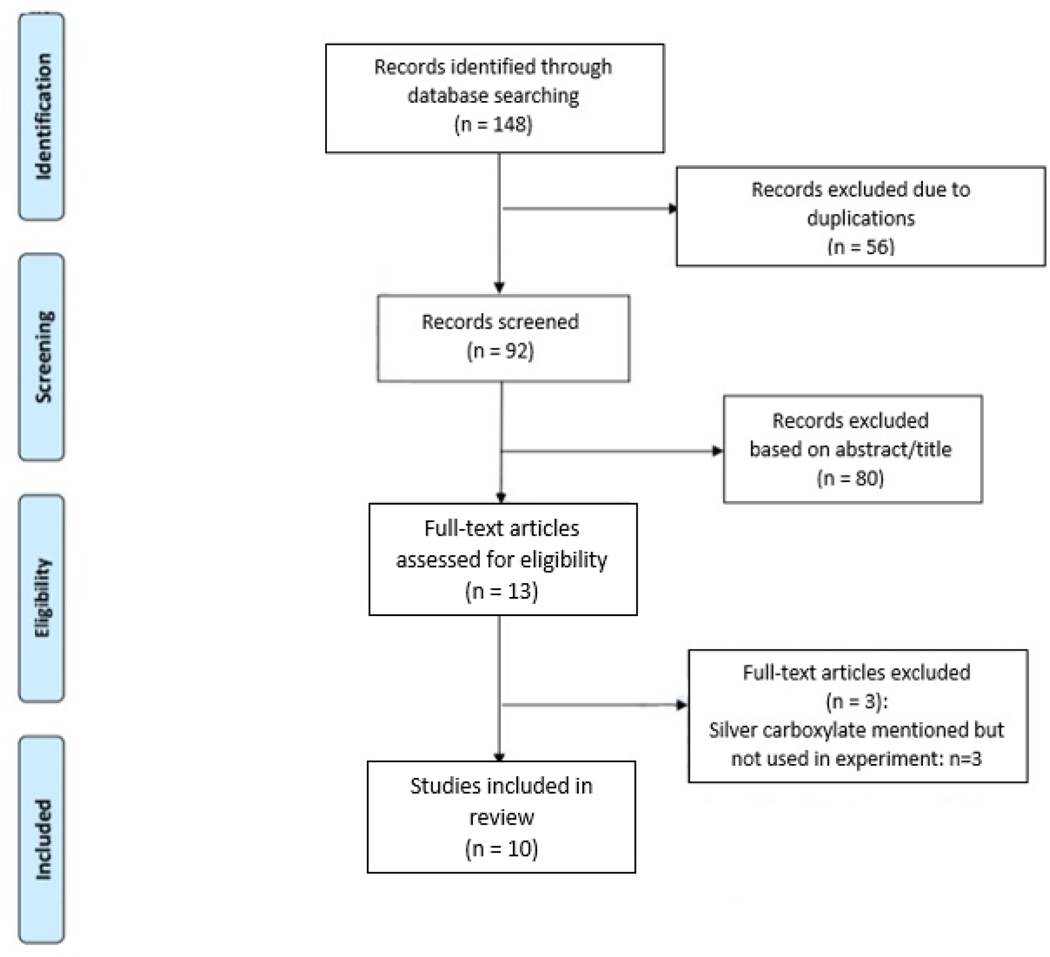
PRISMA Flow Diagram for Study Inclusion

All study designs were deemed eligible, including randomized controlled trials, observational studies, reviews, meta-analyses, case series, and case reports. Studies were deemed eligible for inclusion if they discussed mechanism of action, antimicrobial capacity, or current limitations of silver carboxylate. For each eligible abstract, the full-length manuscript was read and evaluated based on study design and merit. Two reviewers worked independently to determine if a study met inclusion criteria, with the senior author (DRG) providing a final unbiased review when a consensus was not reached. A total of 10 studies were included through this screening process (Figure 1). Articles were compiled, sorted based on silver carboxylate formulation, and then used for draft creation. The results are organized based on formulation.

Silver carboxylate (AgCar) is a metal organic-derived compound that falls into a newer category of antimicrobial delivery systems called metal-organic frameworks (MOFs).^26^ Silver carboxylate is an attractive alternative or synergist to antimicrobials because its chemistry permits a controlled and predictable release of silver, which increases its duration of action while minimizing toxicity risks when compared to previous silver formulations.^29–31^ This predictable and controlled rate of silver ion release is thought to be due to the covalent bond strength between the silver ions and the carboxylated compound.^31^ The ability to control the rate of release allows for the chemistry to be tailored to the specific infection, especially when cytotoxicity may be a concern.^26^

#### Silver Carboxylate (Metal Organic Frameworks) MOFs

AgCar has been shown to be more effective than AgNPs in reducing bacterial growth and proliferation.^33^ Lu *et al.* (2014) synthesized Ag-based metal-organic frameworks from aromatic carboxylic acids with hydroxyl and pyridyl groups as ligands and evaluated their antimicrobial activity using a minimal inhibition concentration (MIC) and inhibition zone testing. This study found that AgCar had a four-fold lower MIC and 50% larger zones of inhibition against both Gram-negative bacteria, *Escherichia coli,* and Gram-positive bacteria, *Staphylococcus aureus,* when compared to silver nanoparticles.^33^ The authors also quantified the release of silver ions in aqueous solution and found that silver MOFs release a greater concentration of silver ions, and that this release is slower and persists longer than AgNPs.^33^ This slow release of silver ions from the MOFs led to effective and long-term antimicrobial activity.^33^ High-resolution transmission electron microscope images demonstrated that MOFs act by rupturing the bacterial membrane, resulting in bacterial death.^33^

The authors also evaluated hematological toxicity of AgCar in the blood cells of mice. When treated with varying concentrations of MOFs at levels that inhibited growth of *E. coli* and *S. aureus*, both total WBC and RBC numbers did not significantly change and there was no significant change in the morphology of the blood cell.^33^ The authors concluded that silver carboxylate MOFs demonstrate significant bactericidal activity without significant cytotoxicity, demonstrating its biocompatibility as an antimicrobial agent.

#### Titanium Dioxide/Polydimethylsiloxane (TiO_2_/PDMS) Matrix Eluting AgCar

Recent work has investigated the efficacy of AgCar in a TiO_2_/PDMS matrix as an antimicrobial coating for commonly used orthopedic implant materials and prosthetic liners.^22,23,29^ Tran *et al.* (2013) initially investigated the cytotoxic effects of silver carboxylate in a titanium/siloxane coating on both *in vitro* osteoblasts and *in vivo* intramedullary nails in a goat model. For the *in vitro* experiments, osteoblast proliferation and viability were assessed using the WST-1 assay (Roche Applied Science, Indianapolis, IN, USA) and *Staphylococcus aureus* growth was assessed via optical density.^32^ *S. aureus* growth was completely eliminated in the presence of 1.8% silver or more in the titanium/siloxane coating, and there was no significant reduction in osteoblast growth until reaching concentrations of 11.36% silver in the coating.^32^ Thus, there was minimal effect on the proliferation of osteoblasts *in vitro* at concentrations that effectively inhibited the growth of *S. aureus.*^32^ The *in vivo* goat study consisted of two goats, each of which was subjected to an open tibial fracture that was inoculated with 2×10^4^ CFU/ml of *S. aureus* and then repaired with an intramedullary nail, either uncoated or with a silver carboxylate - titanium/siloxane coating.^32^ The treated goat lost 7% of its weight compared to 8.4% in the untreated, was better able to ambulate, and was able to remove a fentanyl pain-reliever patch 2 days postoperatively compared to 7 days in the untreated goat.^32^ Based on histological images, the treated goat also was healthier postoperatively, even though both goats still showed signs of *S.aureus* infection.^32^ After 5 weeks, the level of silver detected in either goat’s blood, liver, heart, spleen, brain, kidney, gallbladder, and small intestine was negligible as tested via Inductively Coupled Plasma (ICP) spectrometry, suggesting minimal systemic cytotoxicity.^32^ The authors concluded that there are concentrations of silver carboxylate that effectively inhibit *in vitro S. aureus* growth while displaying no significant osteoblast cytotoxicity, and that *in vivo*, their treated goat was healthier 5-weeks post-operation.^32^ However, this study was limited by its sample size and further research is needed to assess the efficacy of AgCar *in vivo*.

Tran *et al.* (2015) further explored the efficacy of a silver carboxylate titanium oxide-polydimethylsiloxane hybrid matrix on typical orthopedic implant materials. The coatings used in the study benefited from the antimicrobial activity of silver ions, the biocompatibility of titanium dioxide, and the flexibility of the polymer.^30^ The coatings were successfully applied on discs of polyether ether ketone (PEEK), a material used commonly in spinal implants.^30^ The antibacterial property of these coatings was then assessed using Kirby Bauer assays. Three silver doped coatings with different titanium dioxide-PDMS ratios effectively inhibited the attachment and growth of *Staphylococcus aureus* and *Staphylococcus epidermidis* in a dose-dependent manner.^30^ The results of the Tran studies were an important step in the development of silver eluting antimicrobial coatings for orthopedic implants.^30,32^

An additional study by Haglin *et al*. (2020) explored the antimicrobial efficacy and elution qualities of AgCar complexed with a TiO_2_/PDMS matrix on prosthetic liners for limb loss patients who have high rates of skin issues and malodor problems secondary to bacterial colonization. The study utilized dose response curves (DRCs ) and Kirby Bauer assays to explore the antimicrobial efficacy of the silver carboxylate complex and used Graphite Furnace Atomic Absorption Spectroscopy (GFAAS) to perform an analysis of the silver elution on the surfaces of several different commercially available prosthetic liners.^29^ The DRC results exhibited antimicrobial activity with varying degrees of inhibition against vancomycin-resistant *E. faecalis, S. epidermidis,* and *A. baumannii.*^29^ The GFAAS portion of the study also revealed that a 95% 10x solution yielded a significant increase in silver elution over time.^29^ Overall, this study demonstrated promising results for the use of silver carboxylate to mitigate important bacterial pathogen strains, especially *S. epidermidis*, as it is the most common bacteria found on the skin of amputees.^29^ In turn, it may be used on the skin-liner interface of prosthesis-wearing patients with amputation who are at increased risk of bacterial infection.^29^

Garcia *et al.* (2021) investigated the efficacy of a silver carboxylate-doped TiO_2_-PDMS coating against *Serratia marcescens,* a multidrug-resistant gram-negative pathogen increasingly prevalent in spinal SSIs.^22^ PEEK, stainless steel, and titanium (common spinal implant materials) were coated with 95:5 10x silver carboxylate, 100% AgCar, or left untreated.^22^ Adherence was quantified using scanning electron and confocal laser scanning microscopy.^22^ The 95:5 10x silver carboxylate coating reduced the adherence of *S. marcescens* on PEEK by 99.61% (p = 0.001), on titanium by 98.77% (p = 0.001), and on stainless steel by 88.10% (p = 0.001) after 24 hours.^22^ Compared to uncoated implants, the average decrease in bacterial adherence with the 95:5 10x AgCar coating was 95.49%.^22^ Based on these results, the authors concluded that the application of a non-antibiotic, bactericidal coating such as 95:5 10x AgCar prior to spinal surgery implantation may prevent the adherence and proliferation of multidrug-resistant *S. marcescens* and thus decrease the incidence and overall burden of spinal SSI.^22^

Another study by Garcia *et al.* (2022) followed a similar protocol in investigating the efficacy of a silver carboxylate-doped titanium dioxide-polydimethylsiloxane (TiO_2_-PDMS) coating on the adherence and biofilm formation of *Cutibacterium acnes* on PEEK and four other commonly used spinal implant materials, stainless steel, cobalt chromium, titanium, and titanium alloy.^23^ *C. acnes* is a gram-positive biofilm-forming facultative anaerobe found in the deep sebaceous follicles of the shoulder and back, and is gaining increased recognition as a pathogen involved in surgical site infections, especially in the presence of instrumentation.^23^ PEEK, stainless steel, cobalt chromium, titanium, and titanium alloy were either untreated, coated with a 10x AgCar 95:5 TiO_2_-PDMS formulation, or coated with 100% silver carboxylate.^23^ *C. acnes* was then allowed to adhere for 4, 8, 12, 16, or 20 hours. Biofilm adherence and formation were analyzed via confocal laser scanning and scanning electron microscopy.^23^ The 95:5 10x silver carboxylate coating significantly decreased *C. acnes* adherence and prevented biofilm formation on PEEK, both when *C. acnes* was given as little as 8 hours to adhere, or as many as 20.^23^ Furthermore, the 95:5 10x silver carboxylate coating significantly decreased *C. acnes* adherence on all other implant materials, and no biofilm formation was seen on implants with this coating.^23^ This study concluded that a 95:5 10x silver carboxylate coating may serve to decrease the prevalence of SSIs secondary to *C. acnes*, especially in cases using instrumentation.

#### Silver Carboxylate Combined with Chlorhexidine Gluconate (AgCar:CHG)

A recent study published by Garcia *et al.* (2022) analyzed the combined antimicrobial efficacy of silver carboxylate with chlorhexidine gluconate against both methicillin resistant *S. aureus* (MRSA) and *C. acnes*. The authors utilized a Yucatan porcine skin model to demonstrate that this combination coating can penetrate deep into the pilosebaceous gland where *C. acnes* resides at a superior rate to CHG alone.^20^ DRCs for *C. acnes* and MRSA were generated to determine the optimal therapeutic ratio of AgCar to CHG.^20^ Coatings were applied to two different commercially available sutures, Ethicon Polyglactin-910 Coated VICRYL^®^ Plus (Somerville, NJ; termed Ethicon^®^ sutures), Arthrex FiberWire^®^ (uncoated braided polyethylene core structure; Naples, FL; termed FiberWire^®^ sutures), and Ethicon Triclosan Coated VICRYL Plus Antibacterial Sutures (termed Triclosan sutures), and antimicrobial efficacy was evaluated using Kirby-Bauer (KB) assays.20 GFAAS was used to measure AgCar elution from sutures over time.^20^ Sutures coated with AgCar:CHG showed sustained antimicrobial activity against MRSA and *C. acnes*, and were significantly more effective than Ethicon Triclosan-Coated VICRYL Plus Antibacterial sutures (the antimicrobial control) over a three- to four-day period.^20^ These results suggest that the AgCar:CHG complex is a promising additional tool for prophylaxis of surgical site infections; however, more research must be conducted on its cytotoxicity to human cell lines.^31^

#### Silver Carboxylate Colloidal Lignin Particles (AgCLPs)

In Lintinen *et al*’s (2019) publication regarding AgCLPs, the investigators demonstrated antimicrobial efficacy of the silver carboxylate formulation against *E. coli, P. aeruginosa, S. aureus.*^34^ The study demonstrates the AgCLPs strong antimicrobial activity against *E. coli* at 5 mg L–1 Ag+, with 90% inhibition of growth after an exposure of 24 hours.^34^ For *P. aeruginosa*, the Ag+ concentration in AgCLPs required for antibacterial efficiency was 20 mg L–1, leading to an inhibition of 94%.^34^ Lastly, for *S. aureus*, an equivalent of 20 mg L–1 of Ag+ particles was required to inhibit growth of the bacteria by 96%.^34^ The results of this study showed that silver present as a carboxylate is not only stable, it is also strongly antibacterial in physiological conditions with sustained antimicrobial activity.^34^ While this study shows promising results for antimicrobial activity, it does not explore the safety in terms of toxicity to eukaryotic cell lines. Other studies have shown that vehicles are necessary for slow elution of silver carboxylate particles to reduce cytotoxicity.^31^ Further elucidating the cytotoxicity profile of AgCLPs would assist in developing safer formulations for potential silver carboxylate treatments.

#### Silver Carboxylate Complexed with Anti-Inflammatory Ligands

Aldabaldetrecu *et al.* (2018) have investigated silver carboxylate complexes with anti-inflammatory ligands against *Staphylococcus epidermidis*, a multi-drug resistant, biofilm-forming bacterial species found commonly on medical implants, dental photopolymers, and catheters.^31^ These ligands are thought to stabilize the metallic center of the compound and to demonstrate biochemical stability upon exposure to UV radiation.^31^ This is important for topical applications of a silver carboxylate antimicrobial that may be exposed to subsequent radiation therapy treatments.^31^ Transmission electron microscopy demonstrated that these silver carboxylate-anti-inflammatory ligand complexes work via membrane disruption, shedding of cytoplasmic material, penetration of microcrystals, and possible condensation of DNA, preventing DNA replication and cellular reproduction.^31^ The authors found a 100% reduction in *S. epidermidis* after 8 hours of exposure to a 30μg/mL silver carboxylate complex.^31^ Furthermore, the MIC of these compounds against *S. epidermidis* did not significantly increase over a four-week period, showing minimal antibiotic resistance over the course of a four-week treatment.^31^ Finally, an MTT assay was conducted to determine cytotoxicity in fibroblasts and epithelial ovarian cancer cells. Cell viability was not significantly affected at concentrations used in the bactericidal studies mentioned above.^31^ The results support the use of silver carboxylate complexed with anti-inflammatory ligands as a potent antimicrobial, with no significant cytotoxicity in fibroblasts or epithelial ovarian cancer cells at bactericidal concentrations.^31^

#### N-heterocyclic Carbene*Silver Carboxylate Complexes

O’Beirne *et al.* (2021) have devised a formulation of silver carboxylate complexed with N-heterocyclic carbene (NHC). NHC is a ligand that, when complexed with silver carboxylate, allows for biological facilitation of transport and the slow release of silver to cells.^35^ Incorporation of virulence-factor-targeting carboxylate substituents and intracellular protein targeting moieties into the NHC scaffold improves antimicrobial efficacy by directly attacking bacteria.^35^ *In vitro,* NHC*silver carboxylate demonstrated inhibition of growth in *E. coli* (86% reduction in growth)*, Klebsiella pneumoniae* (89%)*, MRSA* (74%)*, Pseudomonas aeruginosa* (87%)*, S. aureus* (73%), and the fungi *Candida albicans* (95%) and *Candida parapsilosis* (77%).^35^ These compounds were then evaluated against biofilms of *P. aeruginosa, MRSA,* and *C. parapsilosis*, and significantly inhibited growth of *MRSA* and *C. parapsilosis*, but not *P. aeruginosa* biofilms.^35^ Finally, an *in vivo* murine thigh MRSA infection model was conducted in mice at doses of 5, 10, 20, and 40 mg/kg.^35^ Although there was a dose-response decrease in CFUs, it was not as pronounced as standard clinical antimicrobials.^35^ Furthermore, at 20 and 40 mg/kgs, there were significant toxicity issues - all test subjects died.^35^ Overall, the authors concluded that these complexes may not be sufficient to clinically treat microbial infections, due to less efficacy when compared to clinical antimicrobials and pronounced toxicity issues at high concentrations, but could be adjuvants to frontline antimicrobial agents.^35^ Moreover, the NHC-silver carboxylate complex demonstrated 100% lethality at concentrations that parallel the antimicrobial efficacy of current clinical antimicrobials^35^; however, an *in vivo* intramedullary nail coated with titanium oxide/siloxane polymer doped with silver did not result in fatality and suggested an improved recovery following tibial fracture, suggesting further comparison of different silver carboxylate modalities are required.^32^

## Conclusion

As antimicrobial resistance and nosocomial infections continue to rise, the development of clinically effective antibiotic-independent antimicrobials, such as silver, has become increasingly important. Current alternatives being explored include the development of novel antibiotics, combination antibiotic therapy, small molecular inhibitors, micro- and nano-technology developments, and the use of indoles, among others. Regardless of formulation, an effective next-generation antimicrobial will extend the shelf-life of current antibiotics through synergistic application, prevent bacterial adherence through compatibility with current antibiotic coatings, augment penetration of antibiotics into inaccessible areas where persister cells and biofilms propagate, and demonstrate bactericidal activity against both persister cells and biofilm dispersion. Silver carboxylate has promise to address each of these challenges. While other silver formulations have clinical limitations due to their negative effects on eukaryotic cell lines and unpredictable pharmacology, existing data on silver carboxylate shows the ability for controlled dosing and lowered potential for cytotoxicity. In comparison to alternative silver-based treatment methods, silver carboxylate’s antimicrobial capabilities and cytotoxicity profile look promising. Approaches such as metal-organic matrixes which control silver carboxylate elution like the silver carboxylate-TiO_2_-PDMS matrix appear to address most of the limitations of other silver formulations. However, more information on cytotoxicity, both *in vitro* and *in vivo* is still needed to fully determine the safety of silver carboxylate formulations in primary human-derived cell lines such as osteoblasts, musculoskeletal, endothelial, erythrocytes, fibroblasts, and keratinocytes. While the cytotoxicity of silver carboxylate was limited in *in vitro* fibroblasts, osteoblasts, epithelial ovarian cancer cells, and red blood cells,^31–33^ *in vivo* efficacy is mixed. As silver interacts with serum proteins, anions, and other sequestrants in the body,^36^ additional *in vivo* tests may show entirely different efficacy and dosing curves, further highlighting the need for more *in vivo* studies of its efficacy.

Although AgCar has shown significant potential as a translational antimicrobial, more research on *in vivo* models is needed to assess its potential for clinical use. Although silver carboxylate formulations show promise as antimicrobial agents, there is a lack of extensive research into their overall efficacy and safety. Therefore, it is imperative that a focus is placed on expanding this body of research to more fully explore the traits of different forms of silver carboxylate treatments, especially given the rise in antibiotic resistance. Furthermore, studies focused on comparing these different modalities may allow for further understanding and combination of different aspects of successful formulations.

## References

[1] Centers for Disease Control and Prevention. National and State Healthcare-Associated Infections Progress Report. Published online 2016. Accessed October 11, 2022. https://www.cdc.gov/hai/data/archive/2016-HAI-progress-report.html

[2] Ban KA, Minei JP, Laronga C, American College of Surgeons and Surgical Infection Society: Surgical Site Infection Guidelines, 2016 Update. J Am Coll Surg. 2017;224(1):59–74. doi:10.1016/j.jamcollsurg.2016.10.029 27915053

[3] Peleg AY, Hooper DC. Hospital-acquired infections due to gram-negative bacteria. N Engl J Med. 2010;362(19):1804–1813. doi:10.1056/NEJMra0904124 20463340 PMC3107499

[4] Young P, Khadaroo R. Surgical site infections. Surg Clin North Am. 2014;94(6). doi:10.1016/j.suc.2014.08.008 25440122

[5] Kern WV, Rieg S. Burden of bacterial bloodstream infection—a brief update on epidemiology and significance of multidrug-resistant pathogens. Clin Microbiol Infect. 2020;26(2):151–157. doi:10.1016/j.cmi.2019.10.031 31712069

[6] Medina E, Pieper DH. Tackling Threats and Future Problems of Multidrug-Resistant Bacteria. Curr Top Microbiol Immunol. 2016;398:3–33. doi:10.1007/82_2016_492 27406189

[7] Ibrahim S, Al-Saryi N, Al-Kadmy IMS, Aziz SN. Multidrug-resistant Acinetobacter baumannii as an emerging concern in hospitals. Mol Biol Rep. 2021;48(10):6987–6998. doi:10.1007/s11033-021-06690-6 34460060 PMC8403534

[8] WHO’s first global report on antibiotic resistance reveals serious, worldwide threat to public health. Saudi Med J. 2014;35(7).

[9] Rather MA, Gupta K, Mandal M. Microbial biofilm: formation, architecture, antibiotic resistance, and control strategies. Braz J Microbiol Publ Braz Soc Microbiol. 2021;52(4):1701–1718. doi:10.1007/s42770-021-00624-x PMC8578483 34558029

[10] Fisher RA, Gollan B, Helaine S. Persistent bacterial infections and persister cells. Nat Rev Microbiol. 2017;15(8):453–464. doi:10.1038/nrmicro.2017.42 28529326

[11] Roy R, Tiwari M, Donelli G, Tiwari V. Strategies for combating bacterial biofilms: A focus on anti-biofilm agents and their mechanisms of action. Virulence. 2018;9(1):522–554. doi:10.1080/21505594.2017.1313372 28362216 PMC5955472

[12] Pang Z, Raudonis R, Glick BR, Lin TJ, Cheng Z. Antibiotic resistance in Pseudomonas aeruginosa: mechanisms and alternative therapeutic strategies. Biotechnol Adv. 2019;37(1):177–192. doi:10.1016/j.biotechadv.2018.11.013 30500353

[13] Coates ARM, Hu Y, Holt J, Yeh P. Antibiotic combination therapy against resistant bacterial infections: synergy, rejuvenation and resistance reduction. Expert Rev Anti Infect Ther. 2020;18(1):5–15. doi:10.1080/14787210.2020.1705155 31847614

[14] Cascioferro S, Carbone D, Parrino B, Therapeutic Strategies To Counteract Antibiotic Resistance in MRSA Biofilm-Associated Infections. ChemMedChem. 2021;16(1):65–80. doi:10.1002/cmdc.202000677 33090669

[15] Rao H, Choo S, Rajeswari Mahalingam SR, Approaches for Mitigating Microbial Biofilm-Related Drug Resistance: A Focus on Micro- and Nanotechnologies. Mol Basel Switz. 2021;26(7):1870. doi:10.3390/molecules26071870 PMC8036581 33810292

[16] Song S, Wood TK. Combatting Persister Cells With Substituted Indoles. Front Microbiol. 2020;11:1565. doi:10.3389/fmicb.2020.01565 32733426 PMC7358577

[17] Kim W, Hendricks GL, Tori K, Fuchs BB, Mylonakis E. Strategies against methicillin-resistant Staphylococcus aureus persisters. Future Med Chem. 2018;10(7):779–794. doi:10.4155/fmc-2017-0199 29569952 PMC6077763

[18] Peng J, Mishra B, Khader R, Felix L, Mylonakis E. Novel Cecropin-4 Derived Peptides against Methicillin-Resistant Staphylococcus aureus. Antibiot Basel Switz. 2021;10(1):36. doi:10.3390/antibiotics10010036 PMC7824259 33401476

[19] Ismat A, Walter N, Baertl S, Antibiotic cement coating in orthopedic surgery: a systematic review of reported clinical techniques. J Orthop Traumatol Off J Ital Soc Orthop Traumatol. 2021;22(1):56. doi:10.1186/s10195-021-00614-7 PMC8702599 34940945

[20] Garcia DR, Vishwanath N, Allu S, Synergistic Effects of Silver Carboxylate and Chlorhexidine Gluconate for Wound Care and Prevention of Surgical Site Infections by Cutibacterium acnes and Methicillin-Resistant Staphylococcus aureus. Surg Infect. 2022;23(3):254–261. doi:10.1089/sur.2021.237 35085476

[21] Eltorai AE, Haglin J, Perera S, Antimicrobial technology in orthopedic and spinal implants. World J Orthop. 2016;7(6):361–369. doi:10.5312/wjo.v7.i6.361 27335811 PMC4911519

[22] Garcia D, Gilmore A, Berns E, Silver carboxylate and titanium dioxide-polydimethylsiloxane coating decreases adherence of multi-drug resistant Serratia marcescens on spinal implant materials. Spine Deform. 2021;9(6):1493–1500. doi:10.1007/s43390-021-00380-w 34173223

[23] Garcia DR, Berns EM, Spake CSL, Silver carboxylate-doped titanium dioxide-polydimethylsiloxane coating decreases Cutibacterium acnes adherence and biofilm formation on polyether ether ketone. Spine J Off J North Am Spine Soc. 2022;22(3):495–503. doi:10.1016/j.spinee.2021.09.011 34666180

[24] Hsu JE, Bumgarner RE, Matsen FA. Propionibacterium in Shoulder Arthroplasty: What We Think We Know Today. J Bone Joint Surg Am. 2016;98(7):597–606. doi:10.2106/JBJS.15.00568 27053589

[25] Caseris M, Ilharreborde B, Doit C, Is Cutibacterium acnes early surgical site infection rate related to the duration of antibiotic prophylaxis in adolescent idiopathic scoliosis surgery? Eur Spine J Off Publ Eur Spine Soc Eur Spinal Deform Soc Eur Sect Cerv Spine Res Soc. 2020;29(7):1499–1504. doi:10.1007/s00586-020-06427-2 32342283

[26] Vishwanath N, Whitaker C, Allu S, Silver as an Antibiotic-Independent Antimicrobial: Review of Current Formulations and Clinical Relevance. Surg Infect. Published online September 29, 2022. doi:10.1089/sur.2022.229 36178480

[27] Lansdown ABG. Silver in health care: antimicrobial effects and safety in use. Curr Probl Dermatol. 2006;33:17–34. doi:10.1159/000093928 16766878

[28] de Lima R, Seabra AB, Durán N. Silver nanoparticles: a brief review of cytotoxicity and genotoxicity of chemically and biogenically synthesized nanoparticles. J Appl Toxicol JAT. 2012;32(11):867–879. doi:10.1002/jat.2780 22696476

[29] Haglin JM, Garcia DR, Roque DL, Spake CSL, Jarrell JD, Born CT. Assessing the Efficacy of a Silver Carboxylate Antimicrobial Coating on Prosthetic Liners. JPO J Prosthet Orthot. 2020;32(4):251–257. doi:10.1097/JPO.0000000000000271

[30] Tran N, Kelley MN, Tran PA, Silver doped titanium oxide-PDMS hybrid coating inhibits Staphylococcus aureus and Staphylococcus epidermidis growth on PEEK. Mater Sci Eng C Mater Biol Appl. 2015;49:201–209. doi:10.1016/j.msec.2014.12.072 25686940

[31] Aldabaldetrecu M, Tamayo L, Alarcon R, Stability of Antibacterial Silver Carboxylate Complexes against Staphylococcus epidermidis and Their Cytotoxic Effects. Mol Basel Switz. 2018;23(7):E1629. doi:10.3390/molecules23071629 PMC6100285 29973523

[32] Tran N, Tran PA, Jarrell JD, *In vivo* caprine model for osteomyelitis and evaluation of biofilm-resistant intramedullary nails. BioMed Res Int. 2013;2013:674378. doi:10.1155/2013/674378 PMC3693125 23841085

[33] Lu X, Ye J, Zhang D, Silver carboxylate metal-organic frameworks with highly antibacterial activity and biocompatibility. J Inorg Biochem. 2014;138:114–121. doi:10.1016/j.jinorgbio.2014.05.005 24935093

[34] Lintinen K, Luiro S, Figueiredo P, Antimicrobial Colloidal Silver–Lignin Particles via Ion and Solvent Exchange. ACS Sustain Chem Eng. 2019;7(18):15297–15303.doi:10.1021/acssuschemeng.9b02498

[35] O’Beirne C, Piatek ME, Fossen J, Continuous flow synthesis and antimicrobial evaluation of NHC* silver carboxylate derivatives of SBC3 *in vitro* and *in vivo*. Met Integr Biometal Sci. 2021;13(2):mfaa011. doi:10.1093/mtomcs/mfaa011 33595656

[36] Alexander JW. History of the medical use of silver. Surg Infect. 2009;10(3):289–292. doi:10.1089/sur.2008.9941 19566416

